# Problem-based learning and larger student groups: mutually exclusive or compatible concepts – a pilot study

**DOI:** 10.1186/1472-6920-8-35

**Published:** 2008-06-18

**Authors:** Martyn P Kingsbury, Joanne S Lymn

**Affiliations:** 1 Centre for Educational Development, Imperial College London, Exhibition Road, South Kensington, London, UK; 2 Faculty of Medicine & Health Sciences, University of Nottingham, Queens Medical Centre, Nottingham, UK

## Abstract

**Background:**

Problem-based learning is recognised as promoting integration of knowledge and fostering a deeper approach to life-long learning, but is associated with significant resource implications. In order to encourage second year undergraduate medical students to integrate their pharmacological knowledge in a professionally relevant clinical context, with limited staff resources, we developed a novel clustered PBL approach. This paper utilises preliminary data from both the facilitator and student viewpoint to determine whether the use of this novel methodology is feasible with large groups of students.

**Methods:**

Students were divided into 16 groups (20–21 students/group) and were allocated a PBL facilitator. Each group was then divided into seven subgroups, or clusters, of 2 or 3 students wh each cluster being allocated a specific case. Each cluster was then provided with more detailed clinical information and studied an individual and distinct case-study. An electronic questionnaire was used to evaluate both student and facilitator perception of this clustered PBL format, with each being asked to rate the content, structure, facilitator effectiveness, and their personal view of the wider learning experience.

**Results:**

Despite initial misgivings, facilitators managed this more complex clustered PBL methodology effectively within the time restraints and reported that they enjoyed the process. They felt that the cases effectively illustrated medical concepts and fitted and reinforced the students' pharmacological knowledge, but were less convinced that the scenario motivated students to use additional resources or stimulated their interest in pharmacology.

Student feedback was broadly similar to that of the facilitators; although they were more positive about the scenario stimulating the use of additional resources and an interest in pharmacology.

**Conclusion:**

This clustered PBL methodology can be successfully used with larger groups of students. The key to success lies with challenging and well situated clinically relevant cases together with enthusiastic facilitators. Facilitator enjoyment of the PBL process may be related to adequate training and previous PBL experience, rather than academic background. The smaller number of facilitators required using this clustered PBL approach allows for facilitators with 'a belief in the philosophy of PBL' to volunteer which would again impact on the success of the process.

## Background

Problem-based learning is recognised as a methodology which promotes integration of knowledge and fosters a deeper approach to life-long learning [1,2]. Although there has been some concern about the value of problem-based learning, over and above lecture-based learning, in terms of knowledge acquisition [3], there have been a number of studies conducted in a variety of countries which indicate that PBL does not disadvantage students [4-8]. Moreover students clearly indicate a preference for this type of learning [5,9-11] and there is some evidence to suggest that medical students following PBL curricula are better disposed towards research [12], and show significant improvements in preventative care and diagnostic performance in practice after graduation [13].

PBL has been described as 'the learning that results from the process of working towards the understanding or resolution of a problem' [14]. While there is no categorical definition of PBL a number of ground rules have been formulated [15]. Although these ground rules suggest that it should be 'curriculum-wide and supported by all curricular elements', PBL is commonly implemented as supplementary to lecture-based learning and/or in a single module of the curriculum [16]. Indeed Kaufmann has argued that we should expect wide variation in the models of PBL implemented with the only key criteria being 'the use of case problems, small group tutorials and self-directed learning activities' [17].

The implementation of this form of teaching however is not without difficulties, a number are inter-related and arise from the long-term running costs, including the number of staff and curriculum hours required to service this model and associated training issues for facilitators [16]. The resource commitment required to utilise this methodology has made it unworkable within a number of institutions, particularly where class sizes are large [1,18]. It has recently been argued that not only is PBL methodology expensive to implement, but is also a misuse of faculty resource and suggests alternative case-based approaches should be developed [19].

From personal experience of teaching pharmacology in a large medical school it was apparent to us that while students were capable of recalling factual information from individual lectures they struggled to integrate knowledge across the wider curriculum. This may be a result of students, faced with an ever-increasing amount of information, particularly in pharmacology [20], adopting a surface rather than a deep approach to learning [1]. Such is the scale of the problem that to quote Dornhorst from over 20 years ago 'all but the brightest students get submerged in the torrent of information' [21].

Medical students need to be able to distinguish pharmacological principles from this information overload, and to understand their relevance, in order to integrate them into clinical situations. According to Kwan 'this is most effectively achieved using a student-centred environment conducive to life-long learning' [22].

While Shanley and others have reported the use of less resource intensive problem based formats to teach large classes [19,23-25], these solutions employ a more structured, didactic approach akin to that described for case-based learning (CBL) [26]. It was important to us however to retain the benefits of the constructive, self-directed, collaborative and contextual processes found in PBL. Hence we designed a novel, clustered, format to accommodate both the large student numbers and the limitations in terms of trained, experienced and willing PBL facilitators.

The overriding aim of this clustered PBL was to use clinically relevant, challenging and realistic problems to provide a scaffold for the integration of previously taught pharmacological knowledge. As such the learning outcomes derived by students from the individual cases, whilst interesting and potentially relevant to other aspects of the medical curriculum were of secondary importance.

According to Norman and Schmidt tutor performance impacts not only on group functioning, but also on both students' perceptions of the quality of the problems used in PBL, and on the amount of prior knowledge needed by the participants [9]. This impact is likely to be even more important when dealing with large groups of students.

The aim of this study was to determine whether the use of this clustered PBL format was feasible with large cohorts of students. Preliminary data from both the facilitator and student perspective on the implementation of this methodology is presented.

## Methods

### Institutional Setting

In a large medical school, with an intake of around 340 medical students per year, basic science concepts remain taught by conventional teacher-centred methodologies. Within this course pharmacology is taught over 21 sessions during the autumn and spring terms of Year 2 and consists mainly of didactic lectures with a small number of tutorials, practical classes and workbook sessions. While the pharmacology and therapeutics component of the undergraduate medical curriculum is delivered mainly through the use of didactic lectures the students are familiar with the process of PBL through the 'Doctor-Patient' course which runs in both the first and second years of the undergraduate curriculum and is delivered using this methodology.

Reorganisation of the 1^st ^and 2^nd ^year medical course resulted in the evolution of a new 'Integrated Body Function and Dysfuction' module to run in the summer term of Year 2. This module was designed to be five weeks long with each week being concerned with a different topic. Bearing in mind our concern regarding the integrative ability of our students we decided to use the pharmacology topic in this module as an opportunity for the students to integrate previously taught pharmacological concepts with pathophysiology in a meaningful context using a student-centred, student-led active learning scenario. Due to the large student numbers involved it was necessary to design a resource-light version of PBL.

### Facilitator Preparation

Eight facilitators, who had previously attended the institutional PBL training course, were recruited from within the Faculty of Medicine. All facilitators had had previous experience of facilitating conventional small-group PBL within the 'Doctor-Patient' course which runs at the beginning of both years one and two. The facilitators did however demonstrate a wide range of experience of PBL facilitation ranging from one term to several years. All facilitators recruited were from a non-clinical academic background being either pharmacologists or physiologists.

In order to prepare for this resource-light PBL format, the scenario and suggested methodology with approximate timings was circulated to all facilitators prior to a team meeting to discuss the cases and process in further detail. The discussion between facilitators at the initial team meeting was recorded in the minutes of the meeting.

### PBL Scenario

The PBL scenario was based on a fictional intensive care unit and featured seven patients admitted with different problems (Table 1). Following discussion with an experienced consultant intensivist an outline sketch of each patient was written by the authors, based on actual cases. The intensivist supplied further detailed clinical information for each patient, again based on current clinical protocols, to ensure that the scenarios were as realistic as possible.

**Table 1:** Brief description of cases used within the clustered PBL scenario.

Case No.	Sketch Outline
1	Respiratory Disease/asthma in an young adult female
2	Depression/possible overdose in an adult male
3	Meningitis in a 7 year old girl
4	Epilepsy following a minor road traffic accident in a pregnant female
5	Liver disease in an alcoholic homeless male
6	Cardiovascular disease in an elderly male
7	Young adult with crush injuries following a road accident

### Clustered PBL format

Medical students are subdivided into small groups of 5–6 students by the University Medical Office on entering the course, this division of students is done alphabetically and students remain in these groups throughout their undergraduate training. These small groups are utilised across the medical curriculum for anything which requires small numbers and are combined in a set format for larger groups. These subgroups were utilised to divide medical students into 16 groups (20–21 students/group) and each group was allocated a PBL facilitator. Each of the eight facilitators was responsible for facilitating two groups of students, one in the morning and one in the afternoon, on both Days 1 and 5.

**Figure 1:**
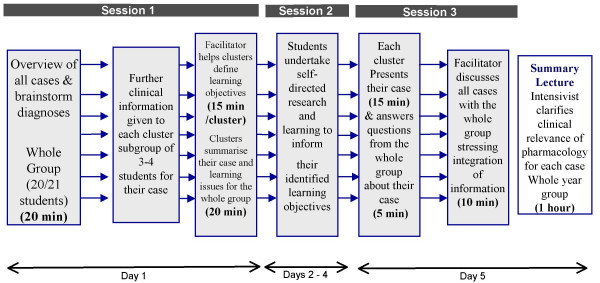
**Clustered PBL methodology**. This figure schematically depicts the process of the resource-light clustered PBL format used.

The overall structure of this clustered PBL methodology is depicted in Figure 1.

Day1 – In the first PBL session each facilitator spent about 20 minutes outlining the context of the PBL and the expectations of the students to their group (20/21 students). Students then received and read through the seven case descriptions supplied, and brainstormed possible explanations, thus all the students were aware of the whole scenario and all of the cases included. The facilitator or a volunteer student recorded the students' initial thoughts on the case studies. At this point each facilitator divided their group (20/21 students) into seven clusters of 2 or 3 students and allocated one of the seven cases to each cluster such that each cluster was allocated a different case study.

Each student cluster then received more detailed clinical information relating to their specific case alone, while the facilitator rotated between clusters spending around 15 minutes with each helping the students to summarise and modify their conclusions and to define relevant learning objectives. The clusters were brought back together at the end of the first session for a 20 minute information and discussion exercise. This brought the whole scenario back together, allowing each cluster to summarise both the extra, detailed, clinical information they received and their learning objectives to the remainder of the group (20/21 students) thus helping the students to appreciate how each case fitted into the overall scenario.

While the cases were chosen to be clinically relevant and challenging, with many potential student defined learning outcomes, the overall learning objective was to encourage the students to integrate previously learned material and demonstrate clinical relevance. The fact that each case and each cluster subgroup had different sets of learning outcomes highlighted the importance of an integrated approach in a variety of clinical circumstances and thus the overall learning objective of integration was emphasised at every stage whilst the students were allowed to follow detailed learning objectives according to their own interests.

Days 2–4 – Students undertook self-directed/collaborative research and learning to inform their identified learning objectives. Two days were timetabled for this self-directed learning during the week but students had unlimited access to appropriate research facilities and could organise their self-directed study as they wished.

Day 5 – In the final session of the PBL each cluster gave a formal, timed presentation to, and responded to questions from their group (20/21 students) in relation to their allocated case. This final session was timetabled for 150 minutes with each cluster having 15 minutes to present their individual case study and learning issues to the group and a further 5 minutes for questions and discussion. This was followed by a brief (10 minute) summary and discussion led by the facilitator to highlight the integrated approach required in each of the seven cases.

All students in the year (approximately 340 students) were invited to attend a summary lecture given by an experienced intensivist to clarify particular points of clinical relevance to each case, reinforce the contextual clinical relevance of the pharmacology content and to further highlight the need to integrate basic science in 'real' clinical situations.

There was no other competing teaching during the 'Integrated Body Function and Dysfunction' course so students had plenty of time to fully engage in the PBL process.

### Evaluation of clustered PBL format

A questionnaire was used to evaluate both student and facilitator perception of this clustered PBL format. Questionnaires were sent electronically to all students and facilitators by the University Medical Office and all replies were received by them before being compiled and forwarded to the authors. This allowed anonymity of student replies to be retained.

The student questionnaire was modified from that originally used by Antepohl and Herzig [5] with students being asked to rate the content, structure, facilitator effectiveness, and their personal view of the wider learning experience. The questionnaires consisted of a single side of A4 paper containing sixteen questions. Fourteen of these questions were answerable using a 5-point likert scale ranging from 1 (strongly disagree) to 5 (strongly agree) while the remaining two questions required the students to indicate which case study they had been assigned to and to rate the level of difficulty of the case study as too complicated, about the right level or too basic.

The facilitator questionnaire contained 19 questions and was based to a large extent on the student questionnaire with 'my/I' being replaced by 'student' in 5 questions.

The remaining 14 questions related to the facilitators perception of their ability to manage the clustered PBL process effectively within the given time constraints along with their enjoyment of the process. Facilitators were also asked to identify the case studies which they felt worked best and least well for both the morning and afternoon groups.

As an evaluation of a novel teaching methodology this study did not require formal ethical approval from the medical school ethics committee. Experimental design and analysis was however performed following the British Educational Research Association's code of ethics (2004) and all data was annonymised before publication.

One of the limitations of this study relates to the timing of the 'Integrated Body Function and Dysfuction' module at the end of the summer term. Although all students received an electronic evaluation questionnaire from the University Medical Office these were sent out after the end of the academic year. Not all students accessed their email account over the summer period and this resulted in a low response rate. Only responses received within a two week period following the end of the academic year were included in the analysis and it must be considered that these responders were students who felt particularly strongly about this teaching methodology. Although the student response rate was relatively poor and may have been biased towards those with strong opinions even this relatively preliminary data was useful in triangulating the views of the tutors with our own views. While the views of the students are obviously important, these are presently being investigated using a more complex study methodology comparing two different student groups. This paper focuses on the views of the tutors to this new teaching and uses the preliminary student data in a supporting role.

### Statistical Analysis

Frequency response of the likert scores were calculated, the relative frequency of scores 1–3, representing disagreement or 'indifference', and scores of 4–5, representing positive agreement with question statement were computed. Median, mean ± SEM score and percentage expressing positive agreement (scoring 4 or 5) are quoted in the text to aid comparison. While we recognise that means are technically not appropriate with this categorical data, they are more fully informative about the data and perhaps of more use for comparing data. Data were cross tabulated in contingency tables and analysed using Pearson's Chi-Square statistic. Students reported measures of facilitator effectiveness were correlated with student enjoyment of the PBL process using Spearman ranked correlation.

## Results

All eight facilitators returned completed questionnaires for analysis and evaluation. Student perceptions of the clustered PBL were obtained from a total of 111 medical students, while this represented approximately 34% of the total year group an accurate percentage cannot be calculated as no record of student attendance was made.

Minutes of the facilitation team meeting revealed that the eight facilitators were, broadly speaking, confident and positive about facilitating this new PBL methodology. They were also very appreciative of the facilitators' notes and the chance to discuss the process in advance. The only issues raised related to possible difficulties in completing the more complex process in the time available. Some facilitators were concerned that the limited time and relatively large group would make it difficult to give adequate time to each cluster. Similarly concerns were raised about completing the presentations in the reporting session. While some of the tutors (3/8) expressed some concerns about not having the clinical experience to be confident in dealing with the complex clinical scenarios, the majority were comfortable facilitating on the basis of the tutors' notes and discussions at the team meeting. Facilitators did not raise any concerns about dealing with conflict or difficult students within the larger group.

### Management of clustered PBL process – timing

Despite the initial misgivings discussed at the pre-facilitation team meeting regarding the likelihood of being able to complete this complex facilitation process within the given time constraints, feedback data showed that, in retrospect, the facilitators did not feel this was a major issue (Table 2). Indeed all facilitators agreed that they had been able to effectively complete the initial session within the time constraints for each of their student groups. With respect to the reporting session all but one facilitator agreed that they had been similarly able to complete this session within the time constraints. The single facilitator who disagreed with this statement reported that while they were successful in this task with one of their groups of students they were unsuccessful with other group. Overall there was a 93.8% agreement that the time constraints did not negatively influence the process.

**Table 2:** Facilitator perception of clustered PBL structure and process

**Statement in questionnaire**	**Median Score**	**Mean ± SEM Score**	**% Positive Agreement (score 4+)**
I was well informed about the structure of the PBL (n = 8)	4.5	4.4 ± 0.26	87.5
I enjoyed facilitating this clustered PBL (n = 8)	4.5	4.4 ± 0.26	87.5
I effectively helped the students towards the learning objectives in the first session (n = 8)	4.0	4.2 ± 0.19	93.8
I effectively completed the first session within the time constraints (n = 8)	5.0	4.9 ± 0.09	100.0
I effectively steered the reporting session to include group discussion (n = 8)	4.0	4.0 ± 0.29	68.8
I effectively completed the reporting session within the given time constraints (n = 8)	5.0	4.4 ± 0.26	93.8

Median and Mean ± standard error (SEM) scores with percentage of facilitators expressing positive agreement (scoring 4 +) on a 5-point likert scale (from 1 = strongly disagree to 5 = strongly agree) in response to questions on PBL structure and process (n = 8).

### Management of clustered PBL process – steering

Facilitators reported that division of the large group into smaller subgroups was largely self-selecting with students naturally forming small groups with people they had worked with before. As a consequence of this facilitators were not required to address or manage conflict between students within a subgroup.

The complexity of the clustered PBL process, with each facilitator rotating around seven different student clusters, each of which is studying a different case, also raised misgivings at the pre-facilitation meeting. A number of facilitators felt that it would be particularly challenging to ensure that all student clusters had been able to discuss their specific case and generate suitable learning objectives. This was perhaps a reflection of the fact that all facilitators were non-clinical members of staff and yet would have to facilitate essentially seven separate and complex clinical cases. In the event only one facilitator disagreed with the statement 'I effectively helped the students towards the learning objectives in the first session', and again this was only true for one of that facilitators' two groups of students.

Steering the reporting session, to include both cluster case presentations and discussion, however proved to be more difficult. Whilst only one facilitator strongly disagreed that they had been able to effectively steer the reporting session for one group of students, four other facilitators 'neither agreed nor disagreed' that their performance was effective for one of their two groups of students. Indeed this questionnaire statement attracted the least support from facilitators; however there was 68.8% agreement suggesting that while this may have been the most challenging stage of the clustered PBL process it was, broadly speaking, managed effectively (Table 2).

### Facilitator evaluation of PBL content

Facilitators were in complete agreement that the content of the PBL scenarios fitted the students' level of knowledge and effectively illustrated medical concepts (Table 3). There was less of a consensus however about whether the PBL scenarios motivated the students to use additional learning resources, with only half of the facilitators agreeing or strongly agreeing with this statement. While only two facilitators (25%) agreed that the PBL scenarios stimulated the students' interest in pharmacology all but one agreed that it had helped to reinforce students' pharmacological knowledge (Table 3).

**Table 3:** Comparison of facilitator and student perception of clustered PBL content and impact on student learning

**Statement in questionnaire**	**Facilitator (n = 8)**			**Student (n = 111)**		
	
	**Median Score**	**Mean ± SEM**	**% Agreement**	**Median Score**	**Mean ± SEM**	**% Agreement**
The content of the PBL scenarios fitted (the students'/my) level of knowledge	4.0	4.4 ± 0.18	100	4.0	4.0 ± 0.08	73.9
The PBL scenarios effectively illustrated medical concepts	5.0	4.6 ± 0.18	100	4.0	4.1 ± 0.08	80.2
The PBL scenarios motivated (students/me) to use additional learning resources	3.5	3.6 ± 0.26	50	4.0	3.9 ± 0.09	69.4
The PBL scenarios stimulated (the students'/my) interest in pharmacology	3.0	3.3 ± 0.16	25	4.0	3.5 ± 0.10	53.6
The PBL scenarios helped to reinforce (students'/my) pharmacological knowledge	4.0	4.0 ± 0.33	87.5	4.0	3.8 ± 0.10	69.4

Median and Mean ± standard error (SEM) scores and percentage expressing positive agreement (scoring 4+) on a 5-point likert scale (from 1 = strongly disagree to 5 = strongly agree) of both facilitators (n = 8) and students (n = 111) in response to questions on PBL content and its impact on learning.

Interestingly, although all case studies were represented at least once in terms of the facilitator's perception of 'the case study that worked best', a single case study (Case number 7) was represented 9 out of 16 times in terms of the facilitators' perception of 'the case study that worked least well'. If case study 7 could be said to be the facilitators' least favourite case, their favourite case could be argued to be case 4 which received 4 out of 16 votes as 'best' case and no votes as 'worst' case (Table 4).

**Table 4:** Facilitator evaluation of case studies

**(i)**								
**Facilitator**	**A**	**B**	**C**	**D**	**E**	**F**	**G**	**H**

'In my opinion the Case Study which worked best was' (Morning Group)	4	6	1	5	4	1	7	1
'In my opinion the Case Study which worked best was' (Afternoon Group)	3	4	1	5	2	3	7	4

**(ii)**								

**Facilitator**	**A**	**B**	**C**	**D**	**E**	**F**	**G**	**H**

'In my opinion the Case Study which worked least well was' (Morning Group)	7	2	5	7	6	7	1	7
'In my opinion the Case Study which worked least well was' (Afternoon Group)	7	1	5	7	7	7	1	7

Table depicting the case studies which each facilitator selected as: (i) 'the case study which worked best' and (ii) 'the case study which worked least well' for each of their two groups of students.

### Facilitator enjoyment of clustered PBL

Facilitators were broadly positive about this clustered PBL methodology with all but one facilitator (87.5%) agreeing or strongly agreeing that they were both well informed about the structure, and had enjoyed facilitating, the PBL. The facilitator who had neither agreed nor disagreed that they had received enough information about the structure of the PBL actually reported enjoying the facilitation experience, whilst a facilitator who agreed strongly that they had been well informed about the structure reported a 'neither agree nor disagree' response in terms of enjoyment (Table 2).

### Student evaluation of PBL content

Student feedback regarding their view of this novel clustered PBL format was both positive and broadly in line with facilitator views (Table 3) in terms of whether the PBL scenarios fitted their level of knowledge, effectively illustrated medical concepts and reinforced or stimulated their interest in or knowledge of pharmacology. While the students were slightly less positive than the facilitators' 100% agreement that the PBL scenarios fitted their level of knowledge and effectively illustrated medical concepts, there was still a high level of agreement at 73.9% and 80.2% respectively. They were also slightly less positive about the cases helping to reinforce pharmacological knowledge, with 69.4% agreeing compared to 87.5% of the facilitators. However, they were more positive about the PBL scenarios motivating them to use additional resources (69.4% agreeing) and stimulating their interest in pharmacology (53.6% agreeing) compared to the facilitators, with 50% and 25% respectively.

Intriguingly, analysis of the preliminary student feedback from individual case studies did not indicate any significant difference in terms of interest (P = 0.499), enjoyment (P = 0.571), illustration of medical concepts (P = 0.641) or reinforcement of pharmacological knowledge (P = 0.341). Moreover students did not significantly differentiate between the cases in terms of the appropriateness of tutor interventions (P = 0.939) or of tutor interest (P = 0.548). It is interesting to note that the two cases where slightly less than half the students agreed that the tutor steered the first session effectively were cases 4 and 7. These were what could be described as the tutors favourite and least favourite cases. There was also no evidence of any difference in student response when compared according to their facilitator, although we accept that with such a limited student response this is not definitive.

Overall the student response to this PBL was overwhelmingly positive with 82% positively agreeing that they were interested in the subject matter. Furthermore, there was a significant positive correlation between student 'enjoyment of' this clustered PBL format and various measures of tutor 'effectiveness' (Table 5).

**Table 5:** Correlation of student enjoyment of clustered PBL with measures of tutor effectiveness.

Student statements	'I enjoyed taking part in this PBL'	'I consider PBL to be an effective way of learning'
'The tutor clearly explained what was expected of me in this PBL session'	r = 0.39 ***	r = 0.25 **
'The tutor steered the group effectively in the first session'	r = 0.37 ***	r = 0.29 **
'The tutor's interventions were appropriate,	r = 0.44 ***	r = 0.29 **
'The tutor conveyed an interest in this PBL session'	r = 0.39 ***	r = 0.26 **

Table summarising the level of significance of Spearman correlation between student 'enjoyment' of the PBL process with measures of tutor effectiveness, as perceived by the students. Level of significance ** p < 0.01, *** p < 0.001

While the student questionnaire did not contain a free text box for comments the anecdotal reporting of this exercise to other module leaders was so positive that it now provides the backbone of the 'Integrated Body Function & Dysfunction' course with all the other teaching on this module providing accessory material to compliment this 'Intensive Care' scenario.

## Discussion

Despite some initial misgivings about the time restraints involved in managing this more complex clustered PBL methodology, facilitators agreed that they managed the procedure effectively and that they enjoyed the process. They felt that the cases effectively illustrated medical concepts and fitted and reinforced the students' pharmacological knowledge but were less convinced that the scenario motivated students to use additional resources, or stimulated their interest in pharmacology. Student feedback was also broadly positive and supported the view of the facilitators; although they were more positive about the scenario stimulating the use of additional resources and an interest in pharmacology. This probably reflects the students' natural desire to appear positive and possibly to some extent the exasperation of the pharmacology tutors at the apparent lack of enthusiasm for their subject displayed by undergraduate medical students.

Overall feedback suggested that we were successful in our attempt to exploit the constructive, self-directed, collaborative and contextual characteristics of PBL with a relatively large group of students using a resource light clustered PBL approach. While the clinical and methodological complexity of the scenario and the clustered approach followed was undoubtedly responsible for the initial misgivings of the tutors, it may also have contributed to the ultimate success of the approach. Indeed the importance of realistic, multidimensional problems to the success of PBL has been widely acknowledged [27-31] with good problems even being associated with improved tutor performance [32]. Thus, while the complexity of the cases we used may have initially worried the tutors, it is likely that this same complexity contributed to the appreciation and success of the learning.

There were no apparent pressures on facilitators to manage group dynamics and this may well be a result of the fact that due to the nature of the groupings, students had been working together since the beginning of their undergraduate course and knew each other well. The small subgroups of 3–4 students were allowed to form naturally, and again this may have contributed positively to the cohesion and cooperation of the students.

Tutors have argued that PBL can sometimes lead students to ritual behaviour that simulates interaction and involvement rather than actually achieving it [33]. The novelty and methodological complexity of this clustered approach may have acted to discourage such strategic ritualistic behaviour. Indeed, as one tutor noted, the large number of cluster groups 'kept both me and the students on our toes'.

In terms of this study all the facilitators were from a non-clinical background and all were staff tutors making them expert tutors according to Matthes et al. [34], although content expertise will obviously differ between individual facilitators, and it may be better to think of them as experienced. While we recognise there is some debate on the influence of content expertise versus facilitation expertise [35-38], content expertise has implications in terms of the facilitation process itself [39,40] and may have played a role in the determination of case study 7 as being that which worked 'least well' from a facilitator viewpoint. This case study contained arguably the least pharmacology and the most physiology and was chosen by the facilitator who was a physiologist as the case study that had 'worked best' for both of their student groups, while the facilitators who felt case 7 had 'worked least well' were pharmacologists and were operating outside of their 'comfort zone'. Interestingly it would seem that while 'comfort zones' clearly impacted on facilitators' perception of the effectiveness of the case studies, this was not the experience of the students. Generally speaking, students felt that facilitators intervened appropriately in the process, demonstrated an interest in the PBL case studies and facilitated the clustered PBL effectively. The students did not single out Case study 7 as being significantly different from any of the other case studies in terms of facilitator interest or appropriateness of facilitator interactions.

A significant correlation between students' perception of tutor effectiveness and a number of tutor behaviours, including those related to both knowledge of subject matter and facilitation skill has previously been documented [41]. Whilst this study did not set out to measure group performance and learning effectiveness directly, our data do show a correlation between 'student enjoyment' of the clustered PBL process and student perception of facilitator enjoyment and interest in the process.

Facilitators were generally positive about their experience of this clustered PBL process. The one facilitator who reported a 'neither agree nor disagree' response in terms of enjoyment, also reported that they had been unsuccessful in both managing to complete the first session and in effectively steering the reporting session for one of their groups. Interestingly this facilitator had the least experience of PBL facilitation. It may be therefore that this facilitator, whilst a very experienced lecturer, lacked the necessary experience to effectively facilitate the group process and that this impacted on their enjoyment of the process. This facilitator reported that they strongly agreed with the statement 'I was well informed about the structure of this PBL' and while this may be a simple reflection of the information they had received regarding the clustered PBL process, it may also be representative of an overestimation of the facilitators' ability to manage the group process. Indeed literature data suggests that level of experience has been shown to impact on facilitators' perceptions of their ability to undertake facilitation duties with less experienced facilitators being more likely to over-estimate their abilities [42]. Lack of facilitation experience, particularly in terms of managing the group process may be a real issue with respect to this more complex clustered PBL format and suggests that facilitator training is important. Although some evidence suggests that facilitators often remain ambivalent about their abilities prior to facilitating their first session, despite their training, once they have started facilitating they become better equipped to manage group process [42]. Perhaps then the facilitators will become better at managing this type of large-group PBL process over time and this increased experience should then result in facilitator confidence and enjoyment with a knock-on positive effect on student interest and enjoyment – a key ingredient for learning.

### Limitations

Although the use of this clustered PBL methodology appeared to be successful it should be noted that this study was conducted in a single institution and with a defined student group. The limited response from student evaluation of the experience has meant that this is only useful for preliminary triangulation of viewpoints and cannot in itself be utilised as data. Following this preliminary study we have obtained funding to further investigate the usefulness and transferability of this approach to teaching larger groups of students and we are currently studying the effectiveness of this methodology, from a student perspective, in two separate student populations based in different institutions. We also recognise that this is largely a qualitative study based on fairly simple feedback data obtained from tutors following their first experience of this new methodology. While we wanted to capture and report this initial response, we would also like to undertake a more detailed interview based qualitative study when the methodology becomes more embedded in the curriculum.

## Conclusion

PBL type methodology can be successfully used with larger groups of students to promote knowledge integration across conventional subject boundaries.

The key to success lies with challenging and well situated clinically relevant cases that integrate the scientific and clinical aspects together with facilitator enjoyment of the PBL process. Facilitator enjoyment may be related to adequate training and previous PBL experience, rather than with facilitator background. The smaller number of facilitators required for this clustered PBL approach not only reduces the resource implications of a more traditional PBL approach, it also allows for facilitators with 'a belief in the philosophy of PBL' to volunteer. This would again impact on the success of the process.

We believe that by using challenging and clinically relevant problems that allow students to follow their own learning outcomes whilst still fulfilling our overarching objective of integration, we have successfully retained some of the benefits of traditional PBL with larger students groups. Integral to this success has been the ability to use a group of facilitators who are enthusiastic and supportive. With increasing pressures on the faculty resource this type of educationally positive compromise is of obvious and increasing importance.

## Competing interests

The authors declare that they have no competing interests.

## Authors' contributions

JSL and MPK conceived of and designed the study. MPK performed statistical analysis of the data. JSL drafted the manuscript. All authors have read and approved the final manuscript.

## Pre-publication history

The pre-publication history for this paper can be accessed here:


